# Diffuse Leptomeningeal Glioneuronal Tumor: A Systematic Review Highlighting Molecular Heterogeneity and Survival Outcome

**DOI:** 10.3390/cancers18060912

**Published:** 2026-03-11

**Authors:** Chaejin Lee, Ki-Su Park, Seong-Hyun Park, Mee-seon Kim, Jeong-Hyun Hwang

**Affiliations:** 1 Department of Neurosurgery, School of Medicine, Kyungpook National University, Daegu 41944, Republic of Korea; cjlee@knu.ac.kr (C.L.); kisupark@knu.ac.kr (K.-S.P.); psh1997@knu.ac.kr (S.-H.P.); 2 Department of Pathology, School of Medicine, Kyungpook National University, Daegu 41944, Republic of Korea; meeseonkim@knu.ac.kr

**Keywords:** diffuse leptomeningeal glioneuronal tumor, leptomeningeal dissemination, BRAF fusion, MAPK pathway, pediatric low-grade glioma, molecular heterogeneity, survival analysis

## Abstract

**Background/Objectives**: Diffuse leptomeningeal glioneuronal tumor (DLGNT) is a rare central nervous system neoplasm characterized by leptomeningeal dissemination and heterogeneous clinical and molecular features. Owing to its rarity, the prognostic relevance of clinical, radiological, and molecular factors remains poorly defined. This systematic review aimed to comprehensively summarize the clinicopathological characteristics, molecular landscape, treatment strategies, and survival outcomes of patients with DLGNT. **Methods**: A systematic literature search was conducted in PubMed, Embase, Scopus, and Google Scholar to identify published cases of DLGNT. Studies reporting individual patient data were included. Clinical, molecular, treatment, and survival data were pooled. Overall survival (OS) and progression-free survival (PFS) were analyzed using the Kaplan–Meier method, with subgroup analyses according to clinical and molecular variables. **Results**: Seventy-five patients were included. Most patients were pediatric, and spinal leptomeningeal dissemination and hydrocephalus were frequent. BRAF alterations, most commonly KIAA1549::BRAF fusion, were frequently identified, although no molecular marker predicted survival. The median OS was 89 months, and the median PFS was 30 months. Surgical resection was associated with significantly longer OS compared with biopsy only, while a trend toward longer PFS was observed. Survival outcomes did not differ significantly according to age group, BRAF status, chemotherapy, or radiotherapy. **Conclusions**: DLGNT is a rare and heterogeneous tumor with variable presentation and prolonged survival in selected patients. Although surgical resection may be associated with improved survival, interpretation is limited by selection bias. No single molecular alteration reliably predicts prognosis, highlighting the need for prospective multicenter studies with standardized molecular profiling.

## Simple Summary

Diffuse leptomeningeal glioneuronal tumor is a very rare brain tumor that mainly affects children and young adults and often spreads along the brain and spinal cord. Because it is uncommon and difficult to diagnose, treatment strategies vary widely and clinical outcomes remain unpredictable. To address this, we reviewed all published cases reported since this tumor was first defined to summarize clinical features, genetic findings, treatments, and survival outcomes. We found that hydrocephalus and spinal involvement were common, and that surgery was associated with longer survival in selected patients. Genetic alterations affecting tumor growth–related pathways were also frequently observed. This summary of current evidence may help clinicians recognize this tumor earlier and consider appropriate management strategies.

## 1. Introduction

Diffuse leptomeningeal glioneuronal tumor (DLGNT) was first introduced as a distinct entity in the 2016 World Health Organization (WHO) classification of central nervous system tumors [1]. In the 2021 update, two methylation subtypes (DLGNT-MC-1 and DLGNT-MC-2) were proposed, with potential prognostic implications [2]. However, methylation profiling is not yet routinely feasible in many clinical settings due to technical and logistical limitations. DLGNT remains a rare tumor with a low incidence, and the available literature is largely limited to individual case reports or small series. The disease may be misdiagnosed at initial presentation, most commonly as infectious meningitis, due to its nonspecific clinical and radiologic features [3,4,5,6,7,8]. Given these challenges, we conducted a systematic review of published DLGNT cases to better characterize their clinical course, imaging features, pathology, molecular findings, and treatment outcomes.

## 2. Materials and Methods

### 2.1. Search Strategy

This systematic review was conducted in accordance with the Preferred Reporting Items for Systematic Reviews and Meta-Analyses (PRISMA) 2020 statement. The completed PRISMA checklist is provided as Supplementary Material. A comprehensive search of the literature was performed using the following databases: PubMed, Scopus, Embase, and Google Scholar. The search covered the period from 1 January 2016 to 4 June 2024, as DLGNT was first classified as a distinct disease entity in the 2016 WHO classification. MeSH terms and keywords including “diffuse leptomeningeal glioneuronal tumor,” “DLGNT,” “DLGT,” and “DLMGNT” were used in the search. Detailed search strategies and queries for each database are provided in the Supplementary Materials (Table S1).

### 2.2. Study Selection

Following the database search, a total of 979 records were identified: PubMed (*n* = 90), Scopus (*n* = 136), Embase (*n* = 175), and Google Scholar (*n* = 578). After removing duplicates, 618 unique records remained. These were independently screened by two authors (C.L. and K.P.) for relevance based on title and abstract. After full-text review, 50 studies comprising 75 individual patients were included in the final analysis.

### 2.3. Eligibility Criteria

Full-text articles were assessed for eligibility based on predefined criteria. Studies were excluded if they were non-original publications, derived from non-peer-reviewed sources, lacked patient-level data, involved non-human subjects, or were not pathologically confirmed as DLGNT. A total of 535 records were excluded based on these criteria. Full-text assessment was conducted on the remaining 83 articles, of which 32 were excluded for reasons including insufficient data (*n* = 21), incorrect diagnosis (*n* = 7), or being review articles without unique patient data (*n* = 5). Discrepancies between reviewers were resolved by consensus.

### 2.4. Data Extraction

**Figure 1:**
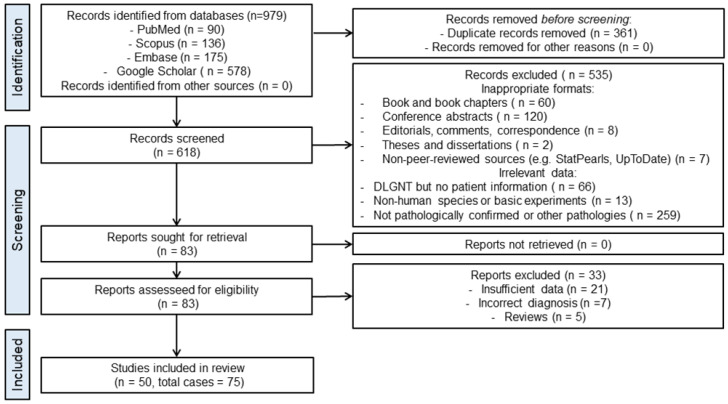
PRISMA flow diagram of study selection process for systematic review of DLGNT cases. Study selection based on case reports and series published between 2016 and 2024.

Data were independently extracted by two authors (C.L. and K.P.), focusing on relevant demographic, clinical, radiological, and histopathological information from the studies. We recorded patient age, sex, presenting symptoms, lesion location, and imaging characteristics. In addition, we collected data on histological and molecular findings (including BRAF status and other mutations), treatment modalities—such as surgery, chemotherapy (CTx), and radiotherapy (RTx)—and survival outcomes, specifically progression-free survival (PFS) and overall survival (OS). The PRISMA flow diagram is presented in Figure 1. Ethical approval was not required for this study, as it was based exclusively on previously published and anonymized data.

### 2.5. Risk of Bias Assessment

The inclusion of only case reports introduces a potential risk of bias. To mitigate this, the quality of the included studies was cross-checked by three authors (C.L., K.P., and S.P.), and the risk of bias was assessed using the Joanna Briggs Institute (JBI) Critical Appraisal Checklist for Case Reports (Appendix A Table A1).

### 2.6. Statistical Analysis

Given the rarity of this pathology, all included studies consisted of case reports or small case series; therefore, the extracted data were primarily summarized using descriptive statistics. Continuous variables are presented as medians with ranges, while categorical variables are reported as frequencies and percentages. Survival outcomes, including OS and PFS, were analyzed using the Kaplan–Meier method. Differences between subgroups were assessed using the log-rank test. A *p*-value < 0.05 was considered statistically significant. All statistical analyses were performed using SPSS version 24.0 (IBM Corp., Armonk, NY, USA) and RStudio (Version 2025.05.1, Build 513; Posit Software, PBC, Boston, MA, USA).

### 2.7. Registration

This review was not prospectively registered. Due to the rarity of the disease and the case-report–based nature of the available literature, no formal review protocol was prepared.

## 3. Results

### 3.1. Patient Characteristics

A total of 75 patients with pathologically confirmed DLGNT were included in this study, spanning 50 published reports [4,5,7,9,10,11,12,13,14,15,16,17,18,19,20,21,22,23,24,25,26,27,28,29,30,31,32,33,34,35,36,37,38,39,40,41,42,43,44,45,46,47,48,49,50,51,52,53,54,55,56]. Among the included patients, 65.3% were male and 73.3% were pediatric (age ≤ 18 years). Initial presentation most commonly included headache (44.0%), nausea/vomiting (36.0%), and seizure (21.3%). Cranial nerve-related symptoms (e.g., visual disturbance, diplopia, hearing loss) were reported in 26.7%. Hydrocephalus was reported in 61.3% of patients, with 45.3% requiring cerebrospinal fluid diversion procedures such as ventriculo-peritoneal shunting (VP shunt) or Ommaya reservoir placement. Notably, 17.3% of patients were initially misdiagnosed, most commonly as infectious meningitis, including tuberculosis.

Brain involvement was observed in 63 patients (84.0%), whereas spinal involvement was present in 46 patients (61.3%). Within the brain, supratentorial and infratentorial involvement were identified in 44 (58.7%) and 50 (66.7%) patients, respectively. Because some patients had lesions in both compartments, these percentages do not sum to 100%. Combined brain and spinal disease occurred in 37 patients (49.3%).

Gross total or subtotal resection was performed in 25.3% of patients, whereas 74.7% underwent biopsy only. CTx was administered in 61.3% of cases, with carboplatin/vincristine (38.7%) and temozolomide (26.7%) being the most commonly used agents. RTx was delivered to 28.0% of patients (Table 1).

A comparative analysis between pediatric and adult patients is presented in Supplementary Table S2. Hydrocephalus was significantly more common in pediatric patients (39/55, 70.9%) than in adults (7/20, 35.0%; *p* = 0.0047), and CTx administration was also more frequent in the pediatric group (40/55, 72.7%) compared with adults (6/20, 30.0%; *p* = 0.0008). In contrast, surgical resection was performed more often in adults (10/20, 50.0%) than in pediatric patients (9/55, 16.4%; *p* = 0.0031).

### 3.2. Molecular and Immunohistochemical Findings

Immunohistochemical analysis showed that synaptophysin and S100 were positive in 44 and 28 cases, respectively, whereas GFAP expression was variable. Olig2 was positive in all 35 cases in which it was assessed.

Pathologic and molecular characteristics are summarized in Table 2. Among chromosomal alterations, 1p deletion was identified in 26 of 30 tested cases (86.7%), while 19q deletion was detected in 15 of 23 tested cases (65.2%). Co-deletion of 1p and 19q was observed in 12 of 23 evaluable cases (52.2%), and 1q gain was identified in two of three tested cases.

**Figure 2:**
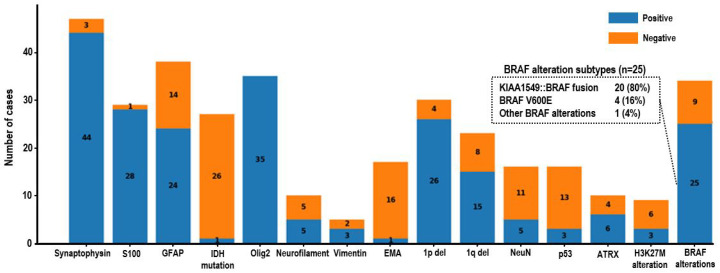
Distribution of immunohistochemical and molecular alterations in DLGNT. Blue bars represent positive findings and orange bars represent negative findings. The inset box summarizes the distribution of BRAF alteration subtypes among the 25 BRAF-altered cases. Values are presented as numbers (%) unless otherwise indicated.

Regarding Mitogen-Activated Protein Kinase (MAPK) pathway alterations, BRAF alterations were detected in 25 of 34 tested cases (73.5%). Among these, KIAA1549::BRAF fusion was the most frequent alteration (*n* = 20), followed by BRAF V600E mutation (*n* = 4), while other BRAF alterations were identified in one case. The distribution of BRAF alteration subtypes is illustrated in Figure 2.

Additional molecular alterations included ATRX mutations (*n* = 6) and H3K27M mutations (*n* = 3). Among the three cases harboring H3K27M mutations, the available reports did not consistently document loss of H3K27me3 immunoreactivity, high-grade histologic features, or clearly worse clinical outcomes; therefore, a definitive association with prognosis could not be established in this cohort. Similarly, methylation subclassification data were available in only three cases (MC-1, *n* = 1; MC-2, *n* = 2), precluding meaningful survival comparisons between methylation classes.

### 3.3. Survival Outcomes and Subgroup Analysis

**Figure 3:**
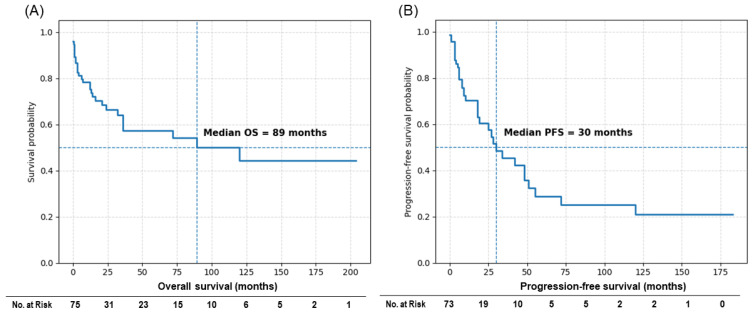
Kaplan–Meier survival curves of the overall DLGNT cohort. (**A**) OS curve for the entire cohort (*n* = 75). The median OS was 89 months. (**B**) PFS curve for the same cohort. The median PFS was 30 months. The number of patients at risk at selected time points is shown below the curves.

**Figure 4:**
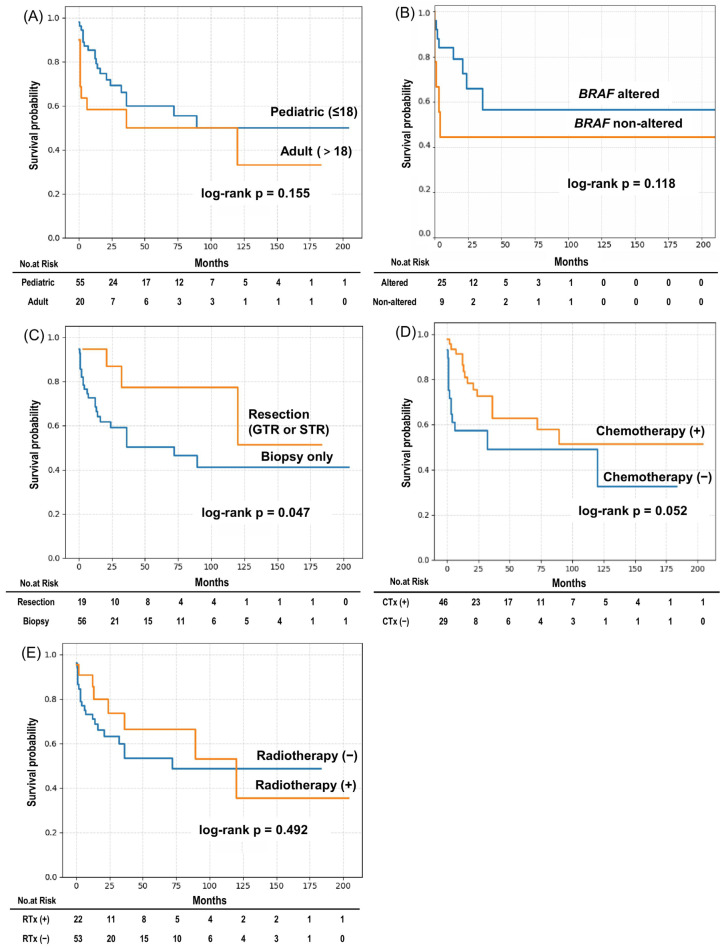
Kaplan–Meier subgroup analysis of OS in patients with DLGNT. Kaplan–Meier curves for OS stratified by: (**A**) Age group (pediatric ≤ 18 years vs. adult > 18 years); (**B**) BRAF alteration status; (**C**) Surgical extent (resection vs. biopsy only); (**D**) CTx; (**E**) RTx. Resection was significantly associated with longer OS (*p* = 0.047), and CTx showed a nonsignificant trend toward longer OS (*p* = 0.052). Other subgroup comparisons did not reach statistical significance. The number of patients at risk at selected time points is shown below the curves.

**Figure 5:**
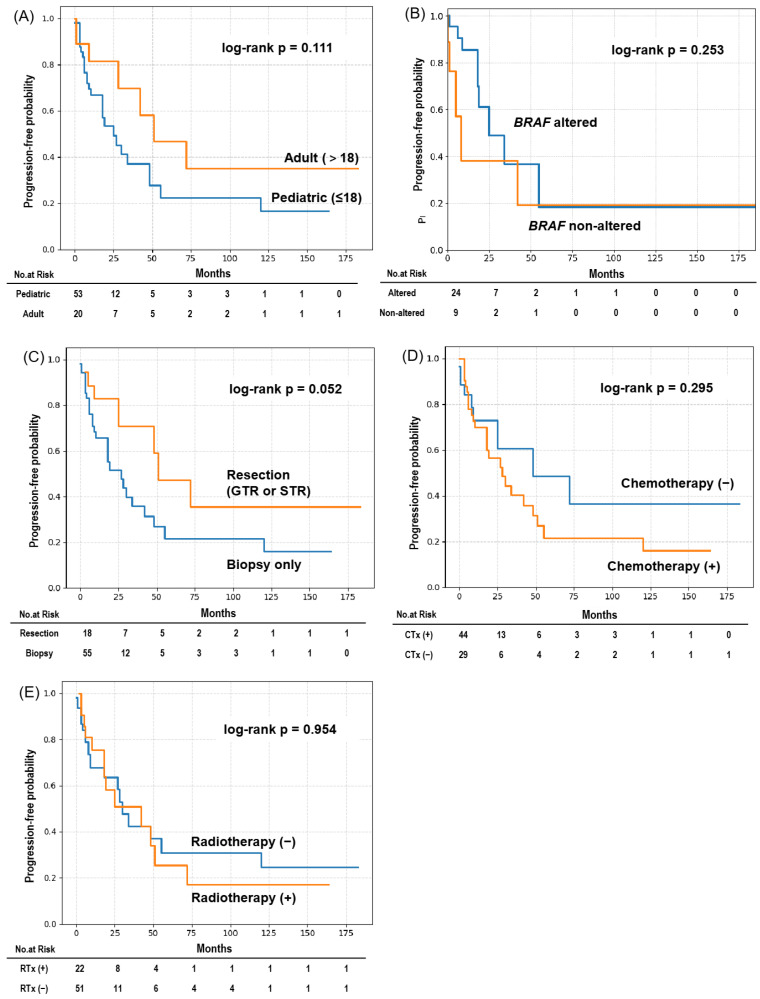
Kaplan–Meier subgroup analysis of PFS in patients with DLGNT. Kaplan–Meier curves for PFS stratified by: (**A**) Age group; (**B**) BRAF mutation status; (**C**) Surgical extent; (**D**) CTx; (**E**) RTx. Patients who underwent surgical resection showed a trend toward longer PFS compared with those who underwent biopsy only (*p* = 0.052). The number of patients at risk at selected time points is shown below the curves.

The median overall survival (OS) for the entire cohort was 89 months, and the median progression-free survival (PFS) was 30 months (Figure 3). Some patients demonstrated prolonged survival beyond 10 years, reflecting the heterogeneous clinical course of this disease. Kaplan–Meier survival curves for OS and PFS according to clinical and molecular subgroups are shown in Figure 4 and Figure 5, respectively, and median survival estimates stratified by subgroup are summarized in Table 3. The number of patients at risk at selected time points is presented in the risk tables accompanying the Kaplan–Meier survival curves. As expected in a cohort derived from heterogeneous case reports, the number of patients at risk decreased progressively over time because of variable follow-up durations and censoring.

There were no significant differences in OS or PFS between pediatric and adult patients (OS: *p* = 0.155; PFS: *p* = 0.111). Similarly, BRAF alteration status was not associated with significant differences in survival outcomes (OS: *p* = 0.118; PFS: *p* = 0.253). BRAF/MAPK-targeted therapies were reported in only a small subset of patients (4/75, 5.3%) and were generally administered in the salvage setting; therefore, the survival analysis according to BRAF alteration status primarily reflects outcomes in patients treated without targeted therapy. Because the survival data were derived from heterogeneous case reports and small case series with variable follow-up durations, these analyses should be interpreted as exploratory.

Patients who underwent surgical resection, including gross total or subtotal resection, had significantly longer OS compared with those who underwent biopsy only (log-rank *p* = 0.047). In terms of PFS, surgical resection was associated with a trend toward longer PFS, although this did not reach statistical significance (*p* = 0.052). However, this finding should be interpreted cautiously because patients undergoing resection likely had more focal or surgically accessible disease, which may reflect lower baseline tumor burden rather than a true treatment effect.

Chemotherapy-treated patients showed a non-significant trend toward longer OS (*p* = 0.052), whereas no significant difference in PFS was observed according to chemotherapy status (*p* = 0.295). Radiotherapy was not associated with significant differences in OS or PFS (OS: *p* = 0.492; PFS: *p* = 0.954).

Additional subgroup analyses according to tumor location and other molecular variables did not demonstrate statistically significant differences in survival outcomes, likely due to the limited number of cases with available data.

## 4. Discussion

In this study, we conducted a systematic review of 75 reported cases of DLGNT in order to summarize the clinical, radiological, pathological, and molecular characteristics of this rare entity. Our pooled analysis demonstrated a predominance of pediatric cases, frequent neuraxis involvement, and a high prevalence of hydrocephalus. Although BRAF fusion was the most commonly reported molecular alteration, no single marker was predictive of prognosis. Surgical resection appeared to be associated with longer OS compared with biopsy-only procedures; however, this observation should be interpreted with caution. Given the diffuse leptomeningeal growth pattern of DLGNT, complete surgical removal is rarely achievable in most patients. Individuals selected for resection likely had more focal or surgically accessible disease, suggesting a lower baseline tumor burden. Therefore, the observed survival advantage may largely reflect selection bias rather than a direct therapeutic effect of surgical resection itself. In addition, survival estimates in the late follow-up period should be interpreted with caution because the number of patients at risk progressively decreased over time. These findings highlight the inherent challenges in interpreting survival outcomes in rare tumors where treatment selection is strongly influenced by disease distribution and surgical feasibility.

DLGNT predominantly affects pediatric patients and often presents with nonspecific neurological symptoms related to intracranial hypertension or leptomeningeal irritation. In our cohort, hydrocephalus was reported in more than 60% of patients, and approximately half of these required cerebrospinal fluid (CSF) diversion procedures such as ventriculoperitoneal shunting or Ommaya reservoir placement. Although these procedures likely contributed to symptomatic relief, their independent impact on survival could not be evaluated. Diagnostic delay is also a recognized challenge in DLGNT [3,6,8]. In our cohort, 17.3% of patients were initially misdiagnosed, most commonly with infectious meningitis, particularly tuberculous meningitis. This diagnostic overlap reflects the nonspecific clinical and radiologic presentation of the disease and underscores the importance of maintaining a high index of suspicion in patients with unexplained chronic leptomeningeal enhancement. Early multidisciplinary evaluation and timely biopsy may therefore be essential for establishing an accurate diagnosis and avoiding prolonged empirical treatment for infectious conditions. Rare presentations of systemic dissemination have also been reported in DLGNT. Although the tumor is generally confined to the central nervous system, extracranial spread to organs such as the lung, bone, and peritoneum has been described in isolated cases [13,20,26], suggesting that systemic dissemination, while extremely uncommon, may occur during the disease course.

Radiologically, most patients demonstrated diffuse leptomeningeal enhancement involving both the brain and spinal cord, frequently accompanied by nodular or microcystic changes along the leptomeningeal surfaces. These imaging features often reflect widespread involvement of the neuraxis and may be associated with communicating hydrocephalus. Less common radiologic findings included intraparenchymal masses and non-enhancing lesions, which have been reported more frequently in the spine than in intracerebral locations. Notably, radiographically apparent leptomeningeal dissemination may not always be identified at the time of initial presentation [57,58]. In some patients, early imaging findings may appear relatively localized before evolving into more diffuse leptomeningeal disease during follow-up. This radiologic variability complicates early recognition and further highlights the importance of integrating imaging findings with histopathological and molecular data in order to establish an accurate diagnosis.

The molecular landscape of DLGNT is characterized by considerable heterogeneity. In our cohort, alterations involving the MAPK signaling pathway were most frequently observed, particularly the KIAA1549::BRAF fusion. These findings are consistent with previous studies demonstrating that DLGNT represents a biologically distinct entity among pediatric glioneuronal tumors, often characterized by the combination of MAPK pathway activation and chromosomal alterations such as 1p deletion [57,59]. Early descriptions by Rodriguez et al. highlighted the distinctive clinicopathologic features of this tumor, and subsequent studies have further refined its molecular classification [58]. These molecular insights have contributed to the recognition of DLGNT as a separate diagnostic entity within the spectrum of pediatric low-grade glioneuronal tumors and emphasize the importance of molecular testing in cases with atypical clinical or radiologic presentations.

More recently, methylation-based classification has provided additional insights into the biological diversity of DLGNT. Deng et al. proposed two methylation subclasses (MC-1 and MC-2), which appear to reflect differences in molecular profiles and potentially clinical behavior [60]. In particular, previous studies have suggested that certain chromosomal alterations may have prognostic implications. For example, 1q gain has been associated with poorer clinical outcomes and appears to occur more frequently in tumors belonging to the DLGNT-MC-2 methylation subclass [61]. These observations suggest that distinct molecular subgroups of DLGNT may exhibit different biological behaviors and clinical trajectories. However, methylation subclassification data were available in only a small number of cases in our dataset, precluding meaningful survival comparisons between subclasses. Recent comprehensive molecular analyses have further highlighted the genomic diversity of DLGNT and reinforced the central role of MAPK pathway alterations in its pathogenesis [62,63,64,65]. These findings emphasize that DLGNT represents a heterogeneous molecular entity within the broader spectrum of pediatric glioneuronal tumors. From a clinical perspective, these molecular insights are particularly relevant, as MAPK pathway activation may represent a potential therapeutic target and provide a rationale for the use of targeted therapies in selected patients. Accordingly, comprehensive molecular profiling may play an increasingly important role in guiding individualized treatment strategies and improving diagnostic accuracy in this rare tumor entity.

Unlike many previous reports that primarily focused on describing the clinical characteristics of DLGNT, our study attempted to explore the potential prognostic relevance of treatment strategies through pooled survival analysis. In our cohort, patients who received CTx demonstrated a trend toward improved survival, although this association did not reach statistical significance. While these findings should be interpreted cautiously, they provide a preliminary signal that treatment-related factors may influence clinical outcomes in this rare disease. The interpretation of treatment effects in DLGNT is inherently challenging, as therapeutic strategies reported in the literature are highly heterogeneous and are often applied in the setting of disease progression or relapse. Consequently, treatment selection may reflect underlying disease severity rather than true therapeutic efficacy. Previous studies have similarly suggested that markers of tumor burden or aggressive tumor biology may influence outcomes. For example, Wiśniewski et al. [66] reported that a high proliferative index, signs of increased intracranial pressure, and neuraxis dissemination were associated with poorer outcomes, while Policicchio et al. [25] identified hydrocephalus and a high proliferative index as adverse prognostic factors. Taken together, these observations suggest that both baseline disease burden and intrinsic tumor biology likely contribute to the variability in clinical outcomes observed in DLGNT. However, the small number of reported cases and the heterogeneity of available clinical and molecular data continue to limit the identification of robust prognostic markers and the establishment of evidence-based treatment strategies.

Currently, no standard treatment strategy has been established for DLGNT. Reported management approaches have typically included combinations of maximal safe resection, craniospinal irradiation, and chemotherapy, although treatment strategies vary widely across institutions due to the rarity of the disease and the absence of consensus treatment guidelines. In clinical practice, treatment decisions are often individualized and influenced by factors such as patient age, disease distribution, symptom burden, and the presence of molecular alterations. Various chemotherapy regimens have been employed, most commonly carboplatin/vincristine–based protocols such as the International Society of Paediatric Oncology Low-Grade Glioma (SIOP-LGG) 2004 regimen or vinblastine-based therapy, which are widely used in pediatric low-grade gliomas and are frequently extrapolated to the management of DLGNT [67,68]. These regimens are generally favored because of their relatively favorable toxicity profile and their established use in other low-grade gliomas. Temozolomide has also been used in some cases, particularly in the setting of progressive or disseminated disease [11,14,20,28,38,39], although the clinical benefit of this approach remains uncertain due to the limited number of reported cases.

Because alterations of the MAPK signaling pathway are common in DLGNT, molecularly targeted therapies have recently emerged as a potential therapeutic option. For example, trametinib has demonstrated clinical activity in patients with BRAF fusion–positive DLGNT [69], and targeted agents such as larotrectinib have shown efficacy in NTRK fusion–positive glioneuronal tumors [70]. Although these reports remain limited to small case series and individual reports, they highlight the potential role of molecular profiling in guiding individualized therapeutic strategies. As molecular diagnostics become more widely implemented, targeted therapies may play an increasing role in the management of selected patients with DLGNT.

This study has several limitations. First, this systematic review was not prospectively registered and no formal review protocol was established. Given the rarity of DLGNT and the predominance of single case reports in the literature, the review process was conducted in a pragmatic manner to capture all available cases. Nevertheless, we acknowledge that this approach deviates from the best-practice recommendations outlined in PRISMA guidelines. Second, most included publications were single case reports or small case series, introducing potential selection and publication biases. Molecular data were incomplete for many patients, limiting robust genotype–phenotype correlations. Survival outcomes were also inconsistently reported, restricting detailed survival analyses beyond median OS and PFS. Because survival data were derived from heterogeneous case reports and small case series, the Kaplan–Meier analyses should be interpreted as exploratory rather than definitive estimates of treatment effect. In addition, treatment regimens were highly heterogeneous across studies. Various chemotherapy protocols—including carboplatin/vincristine-based regimens, temozolomide, and other agents—were grouped together in the analysis due to the limited number of cases available. Given the molecular heterogeneity of DLGNT, such grouping may not fully reflect the differential therapeutic effects of individual agents. Furthermore, tumor size measurements were inconsistently reported across the included studies, and in many cases, the diffuse leptomeningeal growth pattern of DLGNT precludes reliable measurement of tumor size on imaging. Consequently, quantitative comparisons of baseline tumor burden between resected and biopsy-only cases were not feasible. Finally, although our search strategy focused on studies published after 2016—when DLGNT was first recognized as a distinct entity in the WHO classification of central nervous system tumors—earlier cases that were subsequently reclassified as DLGNT were frequently incorporated into later reports. As a result, these previously reported cases were indirectly captured through the included publications.

Despite these limitations, this review provides an updated synthesis of the clinical, radiologic, and molecular characteristics of DLGNT. Future prospective multicenter registries will be necessary to collect standardized clinical, radiologic, and molecular data—including DNA methylation subclassification—as well as treatment responses and long-term outcomes. Such efforts will be essential for improving risk stratification and developing evidence-based treatment strategies for this rare and heterogeneous tumor.

## 5. Conclusions

In conclusion, DLGNT is a rare and heterogeneous central nervous system tumor characterized by frequent leptomeningeal dissemination, hydrocephalus, and diverse molecular alterations. This systematic review provides an updated synthesis of the clinical presentation, radiologic characteristics, molecular landscape, and treatment outcomes reported to date. Our findings highlight the predominance of pediatric cases, frequent diagnostic delay, and the absence of reliable prognostic biomarkers despite the frequent involvement of the BRAF/MAPK signaling pathway. Treatment strategies remain highly heterogeneous across reported cases, and current survival analyses are limited by small sample sizes and inconsistent reporting. Future collaborative multicenter studies integrating standardized clinical data, comprehensive molecular profiling, and longitudinal outcome reporting will be essential to better define prognostic factors and guide more rational, individualized therapeutic strategies for patients with this rare tumor.

## Figures and Tables

**Table 1:** Patient characteristics and treatment courses.

Characteristics	Overall (*N* = 75)	Missing Data (%)
**Demographics**		
Sex, n (%)		1 (1.4)
Male	49 (65.3)	
Female	25 (33.3)	
Age, years, n (%)		0 (0)
>18	20 (26.7)	
≤18	55 (73.3)	
**Clinical presentation, *n* (%)**		8 (10.7)
Headache	33 (44.0)	
Nausea or vomiting	27 (36.0)	
Seizure	16 (21.3)	
Altered mental status	15 (20.0)	
Cranial nerve symptoms †	20 (26.7)	
Motor weakness	16 (21.3)	
Back pain	6 (8.0)	
**Disease burden/Clinical course at diagnosis**		
Hydrocephalus, *n* (%)	46 (61.3)	0 (0)
CSF diversion procedure, *n* (%)	34 (45.3)	0 (0)
Initial misdiagnosis, *n* (%)	13 (17.3)	0 (0)
**Radiologic features**		
Involved lesion, *n* (%)		7 (9.3)
Supratentorial involvement	44 (58.7)	
Infratentorial involvement	50 (66.7)	
Spinal involvement	46 (61.3)	
Combined brain and spinal involvement	37 (49.3)	
Systemic metastasis	3 (4.0)	
Enhancement patterns, *n* (%)		7 (9.3)
Diffuse leptomeningeal enhancement	22 (29.3)	
Focal nodular or microcystic change	12 (16.0)	
Mixed	34 (45.3)	
Calcification, *n* (%)	5 (6.7)	7 (9.3)
Brain parenchymal mass, *n* (%)	23 (30.6)	7 (9.3)
Spine parenchymal mass, *n* (%)	28 (37.3)	7 (9.3)
**Treatment**		
Surgery, *n* (%)		0 (0)
Biopsy	56 (74.7)	
Subtotal resection	5 (6.7)	
Gross total resection	14 (18.7)	
Chemotherapy, *n* (%)	46 (61.3)	0 (0)
Carboplatin/Vincristine	29 (38.7)	
Temozolomide	20 (26.7)	
BRAF/MAPK-targeted therapy	4 (5.3)	
Bevacizumab	3 (4.0)	
Radiotherapy, *n* (%)	21 (28.0)	0 (0)

† Includes ophthalmologic symptoms (e.g., visual disturbance, diplopia), ptosis, facial numbness or weakness, hearing disturbance, etc.

**Table 2:** Pathologic and molecular characteristics.

	Characteristic	Overall
Histopathologic features	Anaplastic features	11 (14.7%)
MAPK pathway alterations	Any BRAF alteration	25 (73.5%)
	KIAA1549::BRAF fusion	20 (58.8%)
	BRAF V600E	4 (11.8%)
	Other BRAF alterations	1 (2.9%)
Copy number alterations	1p deletion	26/30 (86.7%)
	19q deletion	15/23 (65.2%)
	1p/19q co-deletion	12/23 (52.2%)
	1q gain	2/3 (66.7%)
Epigenetic subclassification	MC-1	1/3 (33.3%)
	MC-2	2/3 (66.7%)

Values are presented as number (%) or number positive/number tested (%), as appropriate. Denominators vary because molecular testing methods and reporting differed across the included studies.

**Table 3:** Median overall survival and progression-free survival according to clinical and molecular subgroups.

Characteristic	Median OS (Months)	Median PFS (Months)
Age		
Pediatric	21.0 (12.0–66.0)	10.0 (4.0–24.0)
Adult	10.5 (1.0–51.0)	10.5 (1.8–44.2)
BRAF		
wild-type	15.5 (6.8–63.0)	8.0 (3.0–27.5)
Altered	19.0 (12.0–34.5)	16.0 (9.0–24.0)
Surgical extent		
Resection (GTR/STR)	30.0 (17.0–61.5)	19.0 (9.8–50.2)
Biopsy only	14.5 (3.8–55.5)	8.0 (3.0–21.5)
Chemotherapy		
Yes	26.0 (12.0–72.0)	14.5 (5.8–28.5)
No	12.0 (1.0–30.0)	8.0 (1.0–18.0)
Radiotherapy		
Yes	27.0 (12.0–66.8)	18.5 (6.8–40.5)
No	16.0 (5.0–54.0)	8.0 (3.0–21.0)

Median overall survival (OS) and progression-free survival (PFS) according to clinical and molecular subgroups in patients with diffuse leptomeningeal glioneuronal tumor (DLGNT). Survival outcomes are presented as median (interquartile range), in months. OS and PFS were estimated using the Kaplan–Meier method.

## Data Availability

No new data were created or analyzed in this study. Data sharing is not applicable.

## Supplementary Materials

The following supporting information can be downloaded at: https://www.mdpi.com/article/10.3390/cancers18060912/s1, Table S1: Detailed search strategies used for each database (PubMed, Scopus, Embase, and Google Scholar), including specific queries and the number of records retrieved. Table S2: Comparison of clinical and treatment characteristics between pediatric and adult patients.

## Abbreviations

The following abbreviations are used in this manuscript:

ATRX	Alpha thalassemia/mental retardation syndrome X-linked
BRAF	B-Raf proto-oncogene, serine/threonine kinase
CSF	Cerebrospinal fluid
CTx	Chemotherapy
DLGNT	Diffuse leptomeningeal glioneuronal tumor
FISH	Fluorescence in situ hybridization
GFAP	Glial fibrillary acidic protein
H&E	Hematoxylin and eosin
IRB	Institutional Review Board
JBI	Joanna Briggs Institute
MAPK	Mitogen-activated protein kinase
MRI	Magnetic resonance imaging
NGS	Next-generation sequencing
OS	Overall survival
PFS	Progression-free survival
PRISMA	Preferred Reporting Items for Systematic Reviews and Meta-Analyses
RTx	Radiotherapy
VP	Ventriculoperitoneal

## Appendix A

**Table A1:** Quality appraisal table of included case reports using Joanna Briggs Institute (JBI) checklist for case reports (D1–D8).

	Author, Year	Case Number	D1	D2	D3	D4	D5	D6	D7	D8	Overall Appraisal
1	Al-Ghanem, R. et al. 2022 [9]	1	yes	yes	yes	yes	yes	yes	yes	yes	Include
2	Appay, R. et al. 2020 [10]	1	yes	yes	unclear	yes	yes	unclear	yes	yes	Include
3		2	yes	yes	yes	yes	yes	yes	yes	yes	Include
4	Bao, J. et al. 2023 [11]	1	yes	yes	yes	yes	yes	yes	yes	yes	Include
5	Bao, Y. et al. 2019 [12]	1	yes	yes	yes	yes	yes	Not applicable (refused treatment)	yes	yes	Include
6	Battini, S. et al. 2023 [13]	1	yes	yes	yes	yes	yes	yes	yes	yes	Include
7	Ozen, A. et al. 2024 [14]	1	yes	yes	yes	yes	yes	yes	yes	yes	Include
8		2	yes	yes	yes	yes	yes	yes	yes	yes	Include
9		3	yes	Yes	yes	yes	yes	yes	yes	yes	Include
10		4	yes	Yes	Yes	yes	yes	yes	yes	yes	Include
11	D. V. Davydov et al. 2023 [15]	1	yes	yes	yes	yes	yes	yes	yes	yes	Include
12	Torres-Rey et al. 2023 [16]	1	yes	yes	yes	yes	yes	yes	yes	yes	Include
13	Madsen et al. 2023 [17]	1	yes	yes	yes	yes	yes	yes	yes	yes	Include
14	Lei, X. et al. 2023 [18]	1	yes	yes	yes	yes	yes	Not applicable (refused treatment)	yes	yes	Include
15	Laghaei Fariman et al. 2023 [19]	1	yes	yes	yes	yes	yes	yes	yes	yes	Include
16	Demir, M. et al. 2023 [20]	1	yes	yes	unclear	yes	yes	unclear	yes	yes	Include
17		2	yes	yes	yes	yes	yes	yes	yes	yes	Include
18		3	yes	yes	yes	yes	yes	yes	yes	yes	Include
19		4	yes	yes	yes	yes	yes	yes	yes	yes	Include
20		5	yes	yes	yes	yes	yes	yes	yes	yes	Include
21	Zhang, Z. et al. 2022 [4]	1	yes	yes	yes	yes	yes	yes	yes	yes	Include
22	Yamada, S. et al. 2022 [21]	1	yes	yes	yes	yes	yes	yes	yes	yes	Include
23	Stapińska-Syniec, A. et al. 2022 [22]	1	yes	yes	yes	yes	yes	yes	yes	yes	Include
24	Sarkinaite, M. et al. 2022 [23]	1	yes	yes	yes	yes	yes	yes	yes	yes	Include
25	Rebella, G. et al. 2022 [24]	1	yes	yes	yes	yes	yes	yes	yes	yes	Include
26		2	yes	yes	yes	yes	yes	yes	yes	yes	Include
27		3	yes	yes	yes	yes	yes	yes	yes	yes	Include
28	Policicchio, D. et al. 2022 [25]	1	yes	yes	yes	yes	yes	yes	yes	yes	Include
29	Messiaen, J. et al. 2022 [26]	1	yes	yes	yes	yes	yes	yes	yes	yes	Include
30	Malekian, N. et al. 2022 [27]	1	yes	yes	yes	yes	yes	yes	yes	yes	Include
31	Lu.V.M. et al. 2022 [28]	1	yes	yes	yes	yes	yes	yes	yes	yes	Include
32		2	yes	yes	yes	yes	yes	yes	yes	yes	Include
33		3	yes	yes	yes	yes	yes	yes	yes	yes	Include
34		4	yes	yes	yes	yes	yes	yes	yes	yes	Include
35	Cheng, W. et al. 2022 [29]	1	yes	yes	yes	yes	yes	yes	yes	yes	Include
36	Valiakhmetova, A. F. et al. 2021 [30]	1	yes	yes	yes	yes	yes	yes	yes	yes	Include
37		2	yes	yes	yes	yes	yes	yes	yes	yes	Include
38	Teh, Y.G. et al. 2021 [5]	1	yes	yes	yes	yes	yes	Not applicable (refused treatment)	yes	yes	Include
39	Sáez-Alegre, M. et al. 2021 [31]	1	yes	yes	yes	yes	yes	yes	yes	yes	Include
40	Perez-Vega, C. et al. 2021 [32]	1	yes	yes	yes	yes	yes	yes	yes	yes	Include
41	Peer, S. et al. 2021 [7]	1	yes	yes	yes	yes	yes	yes	yes	yes	Include
42	Manoharan, N. et al. 2021 [33]	1	yes	yes	yes	yes	yes	yes	yes	yes	Include
43		2	yes	yes	yes	yes	yes	yes	yes	yes	Include
44	Karimzadeh P, et al. 2021 [34]	1	yes	yes	yes	yes	yes	yes	yes	yes	Include
45	Xu, H. et al. 2020 [35]	1	yes	yes	yes	yes	yes	yes	yes	yes	Include
46	NJ Marianayagam et al. 2020 [36]	1	yes	yes	yes	yes	yes	yes	yes	yes	Include
47	Gai, D. et al. 2020 [37]	1	yes	yes	yes	yes	yes	yes	yes	yes	Include
48	Chen, W et al. 2020 [38]	1	yes	yes	yes	yes	yes	yes	yes	yes	Include
49	Abongwa C et al. 2020 [39]	1	yes	yes	yes	yes	yes	yes	yes	yes	Include
50		2	yes	yes	yes	yes	yes	yes	yes	yes	Include
51		3	yes	yes	yes	yes	yes	yes	yes	yes	Include
52	Tiwari, N et al. 2019 [40]	1	yes	yes	yes	yes	yes	yes	yes	yes	Include
53	Sasaki, S. et al. 2019 [41]	1	yes	yes	yes	yes	yes	yes	yes	yes	Include
54	Lu, Q et al. 2019 [42]	1	yes	yes	yes	yes	yes	yes	yes	yes	Include
55	Levenbaum et al. 2019 [43]	1	yes	yes	yes	yes	yes	yes	yes	yes	Include
56	Yamasaki, T et al. 2018 [44]	1	yes	yes	yes	yes	yes	yes	yes	yes	Include
57	Nambirajan, A. et al. 2018 [45]	1	yes	yes	yes	yes	yes	Not applicable (refused treatment)	yes	yes	Include
58	Kang, J., H, et al. 2018 [46]	1	yes	yes	yes	yes	yes	yes	yes	yes	Include
59	Fiaschi, P. et al. 2018 [47]	1	yes	yes	yes	yes	yes	Not applicable (refused treatment)	yes	yes	Include
60	Schwetye, K. E. et al. 2017 [48]	1	yes	yes	yes	yes	yes	yes	yes	yes	Include
61		2	yes	yes	yes	yes	yes	yes	yes	yes	Include
62	Karlowee, V. et al. 2017 [49]	1	yes	yes	yes	yes	yes	yes	yes	yes	Include
63	Cai, S.S. et al. 2019 [50]	1	yes	yes	yes	yes	yes	yes	yes	yes	Include
64	JIN Wei, et al. 2018 [51]	1	yes	yes	yes	yes	yes	yes	yes	yes	Include
65	Pellerino, A. et al. 2018 [52]	1	yes	yes	yes	yes	yes	yes	yes	yes	Include
66	Aguilera, D. et al. 2017 [53]	1	yes	yes	yes	yes	yes	yes	yes	yes	Include
67		2	yes	yes	yes	yes	yes	yes	yes	yes	Include
68		3	yes	yes	yes	yes	yes	yes	yes	yes	Include
69		4	yes	yes	yes	yes	yes	yes	yes	yes	Include
70		5	yes	yes	yes	yes	yes	yes	yes	yes	Include
71		6	yes	yes	yes	yes	yes	yes	yes	yes	Include
72		7	yes	yes	yes	yes	yes	yes	yes	yes	Include
73	Dyson K. et al. 2016 [54]	1	yes	yes	yes	yes	yes	yes	yes	yes	Include
74	Fetta A. et al. 2022 [55]	1	yes	yes	yes	yes	yes	yes	yes	yes	Include
75	Guillén Quesada, A. et al. 2018 [56]	1	yes	yes	yes	yes	yes	yes	yes	yes	Include
