# Supplementary material for: Diffuse Leptomeningeal Glioneuronal Tumor: A Systematic Review Highlighting Molecular Heterogeneity and Survival Outcome
Source: Cancers (Basel). 2026 Mar 11;18(6):912. doi: 10.3390/cancers18060912 (PMC13024437; doi:10.3390/cancers18060912)
Supplement: Supplementary file 1 [file cancers-18-00912-s001.zip › cancers-4163530-supplementary/Table S1.pdf]

Table S1. Detailed search strategies used for each database (PubMed, Scopus, Embase, and Google Scholar), including specific queries and the number of records retrieved.

A comprehensive literature search was performed on June 4, 2024, across four databases: PubMed, Scopus, Embase, and Google Scholar. The search strategies and the number of results retrieved from each database are as follows:

Pubmed
Search query: ("diffuse leptomeningeal glioneuronal tumor"[All Fields] OR "DLGNT"[All Fields] OR "DLGT"[All Fields] OR "DLMGNT"[All Fields]) AND (2016:2024[pdat])
Number of records retrieved: 90

Scopus
Search query: TITLE-ABS-KEY (“diffuse leptomeningeal glioneuronal tumor” OR “DLGNT” OR “DLNT” OR “DLMGNT”) AND PUBYEAR > 2015 AND PUBYEAR < 2025
Number of records retrieved: 136

Embase
Search query: ('diffuse leptomeningeal glioneuronal tumor'/exp OR 'diffuse leptomeningeal glioneuronal tumor' OR dlgnt OR dlnt OR dlmgnt) AND [2016-2024]/py
Number of records retrieved: 175

Google Scholar
Search query: “diffuse leptomeningeal glioneuronal tumor”
Number of records retrieved: 578

In total, 979 records were identified through the initial database search.
