# Supplementary material for: Diffuse Leptomeningeal Glioneuronal Tumor: A Systematic Review Highlighting Molecular Heterogeneity and Survival Outcome
Source: Cancers (Basel). 2026 Mar 11;18(6):912. doi: 10.3390/cancers18060912 (PMC13024437; doi:10.3390/cancers18060912)
Supplement: Supplementary file 1 [file cancers-18-00912-s001.zip › cancers-4163530-supplementary/Table S2.pdf]

Table S2. Comparison of clinical and treatment characteristics between pediatric and adult patients.

	**Pediatric (≤18 years, n = 55)**	**Adult (>18 years, n = 20)**	**p-Value**
Male sex	36/55 (65.5%)	13/20 (65.0%)	0.97
Supratentorial involvement	31/55 (56.4%)	13/20 (65.0%)	0.50
Infratentorial involvement	37/55 (67.3%)	13/20 (65.0%)	0.85
Spinal involvement	36/55 (65.5%)	10/20 (50.0%)	0.22
Hydrocephalus	39/55 (70.9%)	7/20 (35.0%)	0.0047 *
Anaplastic features	8/55 (14.5%)	3/20 (15.0%)	0.96
Surgical resection (GTR/STR)	9/55 (16.4%)	10/20 (50.0%)	0.0031 *
Chemotherapy (any)	40/55 (72.7%)	6/20 (30.0%)	0.0008 *
Radiotherapy (any)	14/55 (25.5%)	7/20 (35.0%)	0.42
BRAF alteration (any)	24/55 (43.6%)	10/20 (50.0%)	0.62

Data are presented as number/total (percentage). Comparisons between pediatric and adult patients were performed using the chi-square test or Fisher's exact test, where appropriate. * $p < 0.05$.
